# Endophytic bacteria: a sustainable strategy for enhancing medicinal plant cultivation and preserving microbial diversity

**DOI:** 10.3389/fmicb.2024.1477465

**Published:** 2024-11-18

**Authors:** Giulia Semenzato, Renato Fani

**Affiliations:** Department of Biology, University of Florence, Florence, Italy

**Keywords:** endophytic microbiome, medicinal plants, sustainability, bio-inoculants, secondary metabolites

## Abstract

Endophytic bacteria, part of the plant microbiome, hold significant potential for enhancing the cultivation and sustainability of medicinal plants (MPs). These microbes are integral to many plant functions, including growth promotion, nutrient acquisition, and resistance to biotic and abiotic stresses. However, traditional cultivation practices often overlook the importance of these beneficial microbes, leading to reduced crop yields, lower phytochemical quality, and increased susceptibility to diseases. The domestication of MPs and the use of chemical fertilizers disrupt the natural microbial diversity in soils, essential for the health and productivity of plants. This disruption can lead to the loss of beneficial plant–microbe interactions, which are vital for the production of bioactive compounds with therapeutic properties. Recent advances in microbiome research, supported by omics technologies, have expanded our understanding of how endophytic bacteria can be leveraged to enhance MP productivity and quality. Endophytic bacteria can directly boost MP productivity by promoting plant growth and health or indirectly by restoring healthy soil microbiomes. They can also be harnessed as microbial factories to produce valuable natural compounds, either by transforming plant-derived precursors into bioactive substances or by synthesizing unique metabolites that mimic MP secondary metabolites. This offers a sustainable and low-cost alternative to traditional MP cultivation, reducing the carbon footprint and preserving endangered species. In conclusion, integrating microbiome research with traditional agricultural practices could revolutionize MP cultivation. By focusing on the microbial component, particularly endophytes, we can develop more sustainable and productive methods for cultivating these plants, ultimately contributing to biodiversity conservation and the production of high-value natural products.

## 1 Introduction

Ten percent of all vascular plants have been employed as medicinal plants (MPs) since ancient times in the context of traditional medicine (Salmerón-Manzano et al., 2020; Nath et al., 2023). Nowadays, hundreds of MP species are cultivated worldwide, with Asia and Africa being unsurpassed in the knowledge, cultivation, and production of raw materials and valuable natural products (Salmerón-Manzano et al., 2020).

One of the most valuable products that can be obtained from MPs is the essential oil (EO). Many EOs are renowned for their stress-relieving properties (Matsubara et al., 2023) and have been utilized in various treatments, including those for sleep disorders (Luo and Jiang, 2022), cardiovascular issues (Saljoughian et al., 2018), and cancer (Garzoli et al., 2022). Additionally, they possess antioxidant, anti-inflammatory, antibacterial, antibiotic, and/or antiviral properties (Brochot et al., 2017; Spisni et al., 2020). Essential oils (EOs), classified by the USFDA as “generally recognized as safe” (GRAS) food additives, possess antimicrobial properties and are authorized for inclusion in various foods and beverages (USFDA, 2015)^1^. EOs are complex substances, including hundreds of chemicals, typically obtained by steam distillation of plant material (Ramsey et al., 2020). These secondary metabolites are thought to be the result of natural selection, as they can confer an advantage to the plant, playing a key role in their adaptation to multiple environmental conditions (Carlen, 2016). Plant secondary metabolic pathways are highly complex and strictly regulated by spatial and temporal signals. As might be expected, it is quite expensive for plants to produce these compounds: plants produce secondary metabolites in minimal amounts, mainly due to structural cellular economy and energy budgeting, and their intrinsic cellular toxicity (Jeelani et al., 2018).

Over the past decades, the EO market has become highly active and successful, with an estimated increase to more than 5 trillion US dollars by the year 2050 (Jeelani et al., 2018). With the trend of “return to nature,” the demand for natural products has escalated rapidly (Huang et al., 2015). According to the World Health Organization (WHO), more than 80% of the world's population employs natural herbals to meet their healthcare needs (Wang W. et al., 2020). To keep pace with the strong market growth of MP-derived products, industries must secure adequate supplies of high-quality MP raw materials, where the active principles are available in the desired concentration (Jeelani et al., 2018; Xing et al., 2023). In some cases, plant species containing valuable compounds for humans are available in the wild, but their limited occurrence represents an obstacle to their industrial use (Strzemski et al., 2021). Indeed, the improper overexploitation of plant populations is pushing an increasing number of valuable species to the brink of extinction (Jeelani et al., 2018), with destructive consequences also to their growth environment (Huang et al., 2015).

Moreover, in some cases, the deficiency in MP resources has become a major bottleneck in clinical treatments and new drug development (Huang et al., 2015). Synthetic chemistry often struggles to replicate complex natural compounds, making it an economically unfeasible alternative to the exploitation of MPs (Jeelani et al., 2018), which still remains the most important supply of plant-derived pharmaceuticals (Carlen, 2016). To shrink the gap between the supply and demand for MPs, their large-scale cultivation and the optimization of the production of valuable secondary metabolites should be taken into account (Carlen, 2016; Wang F. et al., 2022).

This review will explore the advantages and disadvantages of the agricultural cultivation of MPs, proposing bacterial endophyte applications as a sustainable alternative to common practices.

## 2 Cultivation strategies of MPs and their impact on secondary metabolites' quality and yield

To meet the ever-increasing demand for MPs, innovative strategies for their conservation and sustainable utilization are needed (Jeelani et al., 2018). The agricultural cultivation of MPs represents a viable alternative to wild harvesting and offers numerous benefits: reliable botanical identification, reduced genetic, phenotypic, and phytochemical diversity, availability of well-defined cultivars tailored to stakeholders' needs, improved conservation practices, less variability of the extracts, and a more consistent supply of raw materials (Carlen, 2016). Agronomic research and development are crucial for enhancing the cultivation of MPs, thereby improving their quality, profitability, and sustainability (Strzemski et al., 2021).

### 2.1 MP domestication and breeding

The process of plant domestication involves the selection, modification, and adoption of wild plant species with desirable traits, releasing pressure on wild flora and critically endangered species (Pérez-Jaramillo et al., 2016). The captive cultivation of MPs can improve the biomass and the quality of the active chemical constituents while obtaining standardized products consistently (Jeelani et al., 2018). In general, domesticated crop plants tend to possess more exaggerated physical traits, simpler morphologies, and modified nutritional content (Chen et al., 2015). As in the case of the MP *Agastache mexicana*, an organoleptic differentiation exists between cultivated and non-cultivated plants (Carrillo-Galván et al., 2020). Morpho-physiological investigations highlighted significant differences in floral, seed, and vegetative characteristics between the two plants. Particularly, cultivated *A. mexicana* has larger leaves and flowers, and a more intense corolla pigmentation, with a sweet anise taste and odor, which is associated with greater effectiveness in alleviating gastrointestinal issues (Carrillo-Galván et al., 2020). Indeed, the phytochemical analysis confirmed that the cultivated variety synthesizes a greater number of aromatic compounds compared to the non-cultivated counterpart (Carrillo-Galván et al., 2020).

On the contrary, crop domestication might also lead to a reduction in the genetic diversity of plant cultivars (Martínez-Romero et al., 2020). During domestication, selection is typically strong and focused on a limited number of traits. Particularly, plants are generally selected to focus their energy mainly on growing (Nerva et al., 2022). This process can impact phenotypic characteristics that are not under selection, although important for survival in the wild. For example, cultivated plants are inferior to their wild progenitors for traits involved in drought tolerance, nutrient acquisition, and resistance to pests and pathogens (Gutierrez and Grillo, 2022). There is accumulating evidence that crop domestication has profoundly altered trophic interactions between plants, insects, and their natural enemies (Chen et al., 2015). Domestication has an overall negative effect on plant resistance to herbivores (Whitehead et al., 2017), due to the changes in morphological traits and levels of secondary metabolites, making domesticated plants a better resource for insects as compared to their wild relatives (Pérez-Jaramillo et al., 2016; Chen et al., 2015). Domesticated plants tend to under-express some secondary metabolites related to biotic stress resistance due to selection of plants with better organoleptic attributes (Ferreira et al., 2023). As an example, the production of the indole-derived class of toxic benzoxazinoids in gramineous plants, highly effective against microbial pathogens, weeds, insects, or herbivores, was substantially altered over the course of domestication (Ben-Abu and Itsko, 2021). In some other cases, domestication led to a decrease in the levels of volatile emissions as compared to wild relatives, which in turn may favor the plant colonization by phytopathogens (Pérez-Jaramillo et al., 2016; Chen et al., 2015). Furthermore, domesticated plants are generally highly nurtured (fertilization and irrigation), a condition that has a great impact on their ability to interact with or adapt to the surrounding environment (Nerva et al., 2022).

Although selection and domestication practices have significantly improved crop yields, they have also contributed to the erosion of plant genetic diversity, prompting cultivators to seek new sources of variability in plants (Gopal and Gupta, 2016; Wang X. et al., 2020). Another valuable cultivation strategy to improve MP yields and productivity is represented by plant breeding. The breeding of new cultivars is a key factor in allowing the modification and adaptation of plant genotypes to the requirements of the stakeholders (Carlen, 2016). A superior MP variety should meet the requirements of good yield, quality, adaptability, and permanence under certain cultivation conditions (Wang W. et al., 2020). Compared to traditionally cultivated crops, the breeding of MPs started relatively late, and research in this area is largely in its early stages. The breeding of MPs is thought to be considerably more complex, as both the growth cycle of the plants and the yield and contents of active ingredients must all be taken into account (Wang W. et al., 2020). However, modern breeding techniques are gradually being applied, and some notable advances have been made (Ramawat and Arora, 2021). Selecting the superior genotypes for cultivation, depending on their potential use, is now feasible, and success is often achieved by a combination of biotechnological methods (Korotkikh et al., 2021; Leontaritou et al., 2021). These approaches are important to increase yield and uniformity as well as to modulate and intensify the secondary metabolite pathways for the enhanced production of bioactive constituents (Jeelani et al., 2018), but also for the elimination of unwanted compounds and increased tolerance against abiotic and biotic stresses (Carlen, 2016), thus increasing the profitability of the production of high-quality MP materials. It should be noted that for the MPs, there is an inverse relationship between the amount of raw material produced and the concentration of biological substances within that material: when plants produce more raw material, the concentration of useful biological substances tends to be lower (Korotkikh et al., 2021). This occurs because these beneficial substances are secondary metabolites, which plants use for their own growth, development, and adaptation to external conditions (Korotkikh et al., 2021).

The primary breeding method involves selecting fine varieties from mixed populations, which involves the identification of existing trait variations and the selection of desired traits. Using individual selection, a valuable variety of oregano was selected based on morphological traits, such as the height of the plants and the color of the flowers; the highest EO yield was obtained from medium-height and low-growing plants (Korotkikh et al., 2021). Additionally, hybrid breeding is another traditional technique that combines the good traits from two or more varieties, relying heavily on parent plant genetics and exploiting hybrid vigor (Zhao et al., 2015). On the contrary, mutation breeding involves the induction of mutations through physical or chemical processes. Radiation can have unpredictable outcomes, such as inhibited plant growth, while chemical mutagenesis is less applied in MP breeding (Lal et al., 2020). Efficient tissue culture systems are essential for genetic manipulation, and several systems have been established for MPs (Wawrosch and Zotchev, 2021). Finally, polyploid breeding uses chromosome doubling to create suitable varieties, leading to positive traits like bigger vegetative organs, resistance to adverse conditions, and high active ingredient content (Sattler et al., 2016). Despite their numerous advantages and diverse applications, the breeding of MPs is still not widespread. Biotechnological advancements, such as next-generation sequencing (NGS) techniques, offer valuable support to breeders by providing genomic and transcriptomic information on MPs. NGS-based DNA barcoding, which targets short genomic DNA regions, facilitates the rapid identification and classification of wild MP populations (Sathishkumar et al., 2015). Additionally, *Agrobacterium*-mediated gene transformation aids in improving MPs by overexpressing key genes in secondary metabolite biosynthesis and downregulating adverse compound-producing genes (Ma et al., 2020). Moreover, the CRISPR/Cas9 genome editing method has emerged as a promising tool for inducing targeted mutations and altering MP biochemical profiles (Guo et al., 2022). CRISPR/Cas9 represents a robust, cutting-edge tool that enables the creation of targeted, heritable mutations at specific locations in the plant genome, significantly expediting the development of desired traits compared to traditional breeding, which is typically long and involves changes across multiple loci (Naik et al., 2022). This system leverages guide RNA (gRNA) and Cas9 protein to form a complex capable of site-specific double-strand breaks (DSBs) in DNA, initiating genomic alterations that are stable and heritable to successive generations (Guo et al., 2022). The CRISPR/Cas9 gene editing system allows precise modifications in plant genomes, fostering desirable traits such as improved quality and yield of secondary metabolites while reducing toxic elements for safer medicinal applications (Naik et al., 2022). Moreover, the CRISPR/Cas9 system has the potential to improve disease resistance and herbicide tolerance, reducing chemical inputs and promoting eco-friendly cultivation practices (Naik et al., 2022). Its applications in medicinal plants are mainly focused on a few model plants with complete genome information and efficient genetic transformation systems, such as *Salvia miltiorrhiza* and *Cannabis sativa* (Zhou et al., 2018; Zhang X. et al., 2021). Finally, plant tissue culture is essential for developing biotechnological techniques, enabling conservation, micropropagation, and rapid propagation of MPs, thus enhancing breeding efficiency (Niazian, 2019).

Although some MPs have been cultivated for several years, only a few of them have been the subject of successful breeding selection. Rapid progress has been made in MP breeding research, resulting in superior varieties with high yield and quality; however, modern biotechnology still faces challenges, including unstable genetic traits and low seedling survival rates, preventing large-scale production. In many cases, breeding techniques have led to degraded and mixed varieties, low yields, and variable active ingredient contents among different strains (Wang W. et al., 2020). Indeed, many traits are regulated by several genes and, for this reason, are hardly transmissible to the progeny in a single crossing event (Nerva et al., 2022). It is estimated that the breeding of a new cultivar might need 5–15 years, depending on the species and the selection criteria: methods to accelerate the breeding procedures must be considered, such as the use of morphological, phytochemical, and genetic markers at a very early stage in the reproduction cycle, increasing the number of generations per year, as well as rapid and cheap measurement methods of target traits (Carlen, 2016). Furthermore, it should be considered that transgenic MPs are still not accepted on the European market (Carlen, 2016).

### 2.2 Continuous cropping and the use of chemical fertilizers

In recent years, with the expansion of the planting area, continuous cropping (CC) of MPs has increased remarkably (Zhang M. et al., 2021). However, CC now represents the primary challenge hindering the survival of MPs (Liao and Xia, 2024). The replant disease or consecutive monoculture problem (CCO) refers to the planting of the same crop on the same land for many years in a row. As the cultivation of MPs requires several years or even decades, the problem of CCO exacerbates. This has resulted in reduced seedling emergence, poor crop yield, poor quality of active ingredients, and increased susceptibility to diseases (Liao and Xia, 2024; Xu et al., 2022). It was evidenced that CCO is involved in 70% of medicinal roots and rhizome herb cultivations, such as ginseng, notoginseng, and *Salvia miltiorrhiza* (Liao and Xia, 2024). This may be connected to soil nutrient deficits, the increased allelopathic autotoxicity of root exudates, the accumulation of fungal pathogens, and imbalances in the soil microbial community (Zeeshan Ul Haq et al., 2023; Wang et al., 2023). Moreover, it may result in photosynthesis changes due to the lack of nitrogen and phosphorus in the soil under CC, which inhibits chlorophyll biosynthesis and restricts photosynthesis (Wang et al., 2023).

The main factors and markers of soil's strength are its carbon contents, organic matter, pH, and concentrations of vital micronutrients and macronutrients, which are necessary for the fertility, quality, functionality, and sustainability of soil; all these parameters might be impacted by CC (Zeeshan Ul Haq et al., 2023). This is the case of the large-scale production of high-quality *Amomum villosum*, which led to a substantial decrease in yield, stem wilt, leaf spot, and fruit rot diseases (Wang B. et al., 2022). The long-term CC of *A. villosum* resulted in distinct changes to soil physicochemical properties and enzyme activities, such as the increase of organic matter, nitrogen, and phosphorus and the decrease of the soil pH and potassium content (Wang B. et al., 2022). *Conodopis tangshen*, a rare plant resource for Chinese Traditional Medicine is no exception in this process (Zhang M. et al., 2021). CC reduced the yield and raw material quality and aggravated its root diseases and insect pest susceptibility, together with the reduction of lobetyolin accumulation, the principal bioactive metabolite responsible for *C. tangshen* medicinal properties (Zhang M. et al., 2021). The continuous cropping severely inhibited the growth of stevia, with reduced plant growth parameters and a lower total amount of valuable secondary metabolites (Xu et al., 2022). Moreover, CC inhibited the growth, yield, and quality of *Polygonatum odoratum*, impacting the synthesis of its phenolic acids (Wang et al., 2023), and decreased the yields of ginseng plants up to 80%−100%, inducing severe root rot illnesses, the falling off of roots, and, in some cases, plant death (Zeeshan Ul Haq et al., 2023).

The same plant's development and growth might also be prevented by allelochemicals, a phenomenon known as autotoxicity. The inhibition of plant growth due to autotoxicity events might be caused by interference with respiration and photosynthesis, the antioxidant and growth regulator systems, the restraint of water and nutrient uptake, and the inhibition of cell division and elongation and nucleic acid and protein synthesis and metabolism (Huang et al., 2019). The aqueous extracts from rhizomes, shoots, soil extracts, and rhizome exudates from *Angelica sinensis* caused a significant inhibition in plant seed germination and seedling growth, thus suggesting the great impact of autotoxicity in CC soil (Xin-hui et al., 2015).

The effects of CC are strengthened by anthropogenic factors, which contribute to the worsening of MP cultivation. The increasing global temperature due to greenhouse gas emissions led to an increase in drought stress and water shortage in the agricultural sector (Amani Machiani et al., 2023). Nutrient accessibility for plants is reduced in these conditions because of the reduction of mineral diffusion in the cultivated soil (Amani Machiani et al., 2023). In such a situation, when plant growth is adversely affected, the initial remedy often considered by cultivators is the application of fertilizers. In recent years, we assisted in the increasing employment of chemicals to overcome problems related to pests and diseases, vulnerability to abiotic stress, and nutrient depletion, thus enhancing MP growth and yield (Pérez-Jaramillo et al., 2016). One of the basic yield-generating elements is nitrogen, the most common agent applied in agriculture and horticulture, able to affect the levels of many other metabolites (Strzemski et al., 2021). In the case of MPs, diverse are the responses observed after nitrogen-based fertilizer application: (i) a positive influence on the raw material yield, EO content, and EO yield; (ii) a positive impact on the chemical composition and yield of EO, but no influence on raw material yield; (iii) a positive impact on the raw material yield, but a negative impact on the EO concentration. In the case of the flower heads of the MP *Arnica chamissonis*, there was a positive and statistically significant effect of nitrogen fertilization, soil type, and their interaction on the crop yield, EO content, and EO yield, which was dose-dependent (Sugier et al., 2019). There were also significant qualitative and quantitative differences in the chemical composition of the EO in plants exposed to nitrogen fertilization, resulting in a higher yield of some volatile components, including alpha- and beta-pinene, cumene, germacrene D, spathulenol, decanal, and caryophyllene oxide (Sugier et al., 2019). In *Salvia* spp., higher nitrogen levels significantly increased the contents of tanshinone I, tanshinone IIA, cryptotanshinone, and salvianolic acid B (Xing et al., 2023). The application of ammonium sulfate maximized herb, leaves, and oil yields of *Ocimum amercianum* L. var. *pilosum* (Omer et al., 2008). In another case, nitrogen fertilization improved the yield of above-ground and leaf fresh biomass, leaf EO concentration, and yield, but it did not affect plant morphological traits or the height of basil plants, indicating an enhancement in oil biosynthesis (Sifola and Barbieri, 2006). Contrarily, thyme herb crop yield was improved with the increase of nitrogen fertilizers, but their effect on the EO content was not considerable (Baranauskiene et al., 2003). Furthermore, the excessive use of high doses of nitrogen fertilization negatively impacted the growth of *Carlina acaulis* plants and the biomass yield, while it did not affect the level of the secondary metabolites (Strzemski et al., 2021).

Despite the variable effects of nitrogen fertilization on cultivated MP yield and its impact on the synthesis of EOs and other important secondary metabolites, it is worth considering that these methods often contribute to soil pollution and might result in the excessive presence of chemical residues in MP-derived products (Liao and Xia, 2024). Excessive fertilization can lead to negative effects on the environment, changing soil pH and micronutrient contents (Strzemski et al., 2021; Wang B. et al., 2022). Those chemicals are known to remain in the soil for long periods, converting themselves into toxic and harmful transformation products (Wang B. et al., 2022). From the perspective of achieving more sustainable cultivation and harvesting of MPs, the use of new eco-friendly fertilizers together with the application of intercropping strategies should improve the quantitative and qualitative characteristics of MPs, still preserving ecosystem balance and avoiding the leakage of toxic chemicals in the environment (Amani Machiani et al., 2023). Intercropping is an agricultural practice that involves growing two or more crops in proximity within the same field, maximizing the utilization of available resources, improving crop productivity, and promoting soil health. Intercropping peppermint with soybean resulted in peppermint growth and EO yield increases, compared to sole peppermint cultivation (Maffei and Mucciarelli, 2003). Moreover, the higher percentages of menthol and lower percentages of menthofuran and menthyl acetate improved the quality of the EO (Maffei and Mucciarelli, 2003). Dragonhead plant height and its EO content were positively and significantly influenced when intercropped with fenugreek (Nasiri, 2021). The higher production of dragonhead EO was attributed to the nitrogen-fixing ability of fenugreek, which provided better access to nitrogen (Nasiri, 2021).

Overall, soil amendments, crop rotation, and intercropping may help to overcome the obstacles associated with CC of MPs (Huang et al., 2015). By horizontally planting different plant species with contrasting nutrient requirements, it is possible to mitigate nutrient imbalances and suppress disease occurrence, together with the reduced application of inorganic fertilizers, avoiding the disruption of the ecological environment (Liao and Xia, 2024).

## 3 Plant-associated bacteria

Bacteria are essential for ecosystem functioning and known for beneficial interactions with other microbes as well as macroorganisms (Berg et al., 2020). New sequencing technologies generated a huge amount of data that have highlighted both the ubiquity of microbial communities in association with higher organisms and the critical roles of microbes in maintaining their health (Berg et al., 2020).

It has been estimated that virtually all plant species contain several different endophytic bacteria and fungi. Plant growth-promoting bacteria (PGPB) are typically present as free-living bacteria in the soil (Narayanan and Glick, 2022). Endophytic PGPB are attracted to a specific plant's root exudates and enter the plant through the roots; many of these bacteria are motile and can travel through the plant toward other tissues such as leaves and stems, where they are generally found in lower concentrations than in the plant roots (Bulgarelli et al., 2013). Bacterial endophytes can grow inside plant tissues in a mutualistic relationship with the plant without harming or inhibiting its growth (Schulz and Boyle, 2006). The species- and habitat-specific plant microbiota contributes to multiple aspects of the functioning of the plant holobiont, such as (i) seed germination and growth, (ii) nutrient supply, (iii) resistance against biotic and abiotic stress factors, and (iv) production of bioactive metabolites (Berg et al., 2020).

The understanding of a plant as no more an individual but a larger genetic entity comprising its associated microbial genome, the microbiome, has given rise to the “holobiont” concept. A “holobiont” is an assemblage of a host and its symbionts living and functioning as a unit of selection (Lyu et al., 2021). Host–microbe coevolution leads to specific microbiomes associated with plants (Berg et al., 2020). Potentially, the host and its microbiome can be subject to community selection, which may lead to the evolution of the community itself (Soldan et al., 2022). Through community evolution, specific microbial community phenotypes might arise, which may provide positive microbe-to-host effects (Soldan et al., 2022). From an ecological perspective, the plant holobiont, and not the plant as an individual, is now recognized to respond to the various biotic and abiotic perturbations in a given environment (Gopal and Gupta, 2016). A significant proportion of the plant holobiont's response is due to the microbial symbionts *via* their ecological services of nutrient mineralization and delivery, protection from pests and diseases, and tolerance to abiotic stress (Gopal and Gupta, 2016). The establishment of beneficial interactions with microbes underlies the adaptation of plants to specific and often challenging environmental conditions (Ferreira et al., 2023). However, plant microbiomes are not static. They can dynamically change in response to environmental stimuli, including both abiotic and biotic factors (Pang et al., 2021).

### 3.1 Impact of cultivation on microbiome diversity

As illustrated in the previous paragraphs, domestication, breeding, the use of chemical fertilizers, and intensive cultivation of MPs have led to the appearance of several qualitative issues, such as reduced nutrient use efficiency, increased susceptibility to pests and diseases, inability to overcome abiotic stresses, and lower yields of plants secondary metabolites. Plant microbiota contributes to all these aspects to ensure the proper functioning of the plant; therefore, it is quite possible that the impact of cultivation practices on MP productivity might reflect changes in microbial community composition and functions, hampering the essential interactions that make wild species more resilient to biotic and abiotic stresses (Nerva et al., 2022; Pérez-Jaramillo et al., 2018). If that is the case, it might be expected that cultivated plant-associated microbiota would differ from one of their wild counterparts. Recent studies have found significant differences between the microbiome of commercial genotypes with that of their relative wild types (Nerva et al., 2022). Wild plants have co-evolved over time with the microbial community of native soils, selectively recalling plant-beneficial microbiota from the surrounding environment as their partners (Pérez-Jaramillo et al., 2016). This beneficial association was disrupted by the development of agriculture.

Domestication and breeding activities can significantly shape host microbiomes to a stronger extent than expected. Cultivation has inevitably changed the ecological context of plants grown in the wild, especially to what concerns the soil, which represents the main source of PGPB (Gopal and Gupta, 2016). The abundance of specific taxa varies across space and time; thus, environmental variation in the potential microbial source community can contribute to variations in plant microbiome composition (Gutierrez and Grillo, 2022). It was evidenced that the rhizosphere bacterial community of ancient plant varieties strongly differs from that of modern cultivars, suggesting that wild relatives could establish beneficial interactions with microbes with a higher frequency as compared to domesticated cultivars (Pérez-Jaramillo et al., 2016; Germida and Siciliano, 2001). On the contrary, domesticated plants tend to be less dependent on microbial symbionts than their wild ancestors, thus representing weaker drivers of the assembly of the beneficial plant microbiome (Ferreira et al., 2023).

Moreover, domestication and especially modern breeding techniques have led to the reduction of MP genetic diversity, selecting against certain plant phenotypes if those traits were unsuitable for an agricultural ecosystem (Soldan et al., 2022). Differentiation between wild and cultivated plant microbiomes may arise due to domestication-induced changes in phenotypes that influence host–microbiome interactions (Gutierrez and Grillo, 2022). As an example, root exudates can be reduced in domesticated plants in terms of their abundance or complexity (Gutierrez and Grillo, 2022). After entering the rhizosphere and soil, most exudate compounds may be quickly utilized by soil microbes, recalling beneficial bacteria and/or suppressing detrimental ones (Pang et al., 2021). Different rhizodeposits can influence the rhizosphere microbiome composition and affect the assembly of the root microbiome and the endophytic communities (Pang et al., 2021; Pérez-Jaramillo et al., 2018). Therefore, changes in root architecture and exudation induced by domestication and crop genetic variation may lead to novel microbial associations in the rhizosphere, with the consequent shift in microbial functional traits (Martínez-Romero et al., 2020; Yue et al., 2023). The analysis of the predictive functional profiles of the endophytic bacterial communities in wild and cultivated *Salicornia europaea* plants revealed a significant shift in pathways involved in plant–microbe interactions (Ferreira et al., 2023). Specifically, the bacterial communities associated with wild plants were enriched in bacterial secretion systems, nitrogen metabolism, and carbon fixation, suggesting the recruitment of bacterial symbionts beneficial for plant growth and nutrition (Ferreira et al., 2023). On the contrary, the endosphere microbiome of crop plants was enriched in orthologs related to membrane transport systems and sulfate metabolism, xenobiotic degradation, and pathogenesis, indicating that domestication may also enhance the risk of disease (Ferreira et al., 2023).

Alterations in the structure of rhizosphere microbial communities might arise as a consequence of successive cultivation of MPs. Some secondary metabolites are secreted by MPs and can accumulate in the soil, selectively inhibiting or promoting different microbial species; beneficial microorganisms may decline, favoring the proliferation of pathogenic ones (Zeeshan Ul Haq et al., 2023). The continuous cropping of *Amomum villosum* markedly changed the bacterial and fungal community composition, structure, and diversity (Wang B. et al., 2022). This change was induced by changes in the physical and chemical properties of the soil, such as hardening, salinization, reduced air permeability, imbalances in nutrient levels, and changes in enzyme activity (Liao and Xia, 2024; Misra et al., 2019). It has been evidenced that CC affects the diversity rather than the richness of soil bacteria. The imbalance of bacterial community (and its associated function) may be reflected in the growth inhibition of crops and the lowering of valuable secondary metabolite content, as evidenced for *Stevia rebaudiana* Batoni (Xu et al., 2022). Despite changes in soil physiochemical properties, MPs can release allelopathic toxic substances, inhibiting both the plant itself and the microbial communities residing in the soil. Allelopathic toxic substances in the root exudates of *Panax notoginseng*, such as phenolic acids, inhibited the germination of seeds and reduced the stress resistance of the same plants (Bao et al., 2022). Moreover, phenolic acids significantly changed bacterial community structure (Bao et al., 2022). Toxic compounds in replanted soil of *Angelica sinensis* significantly decreased the number of bacterial functional groups, suggesting that the autotoxic compounds not only affected the plant growth directly but also indirectly by changing the composition and structure of the soil microbial population with the resulting prevalence of pathogenic strains (Xin-hui et al., 2015). In the case of aromatic plants, a considerable fall of EO-bearing leaves could greatly affect the microbial diversity, due to the antimicrobial nature of the EOs (Misra et al., 2019). This was evidenced for the CC of *Mentha* spp., resulting in lower microbial diversity in the surrounding soil (Misra et al., 2019). Bacterial diversity, and so the health and productivity of MPs, could be restored through intercropping practices (Wang F. et al., 2022). In general, it was observed that beneficial bacterial abundance increased with the increase of crop rotation cycle (Wang F. et al., 2022). Intercropping turmeric and ginger with patchouli improved soil microbial abundance, diversity, and community structure by boosting the number of dominant bacteria and improving soil bacterial metabolism and enzymatic activity (Zeng et al., 2020). This was reflected by changes in the soil physical and chemical properties, such as pH and exchangeable minerals (Zeng et al., 2020).

The effects of CC on plant microbiome need to be interpreted in the context of agricultural environments, in which agricultural inputs such as fertilization can have a considerable impact on the host-associated microbiome (Soldan et al., 2022). Excessive usage of chemical fertilizers can lead to alterations in soil physical and chemical properties, depletion of trace elements, and accumulation of heavy metals (Liao and Xia, 2024). This imbalance affects both taxonomical and functional profiles of rhizosphere microbial communities (dysbiosis), leading to an upsurge in soil-borne pathogens (Liao and Xia, 2024; Berg et al., 2020; Soldan et al., 2022). For example, long-term nitrogen addition has resulted in the recruitment of less functional rhizobacteria in leguminous species, providing fewer benefits to the host plant (Pérez-Jaramillo et al., 2016; Nerva et al., 2022; Gutierrez and Grillo, 2022). Studies have shown that the excessive application of nitrogen potash induced salt accumulation and decreased the C/N ratio, resulting in changes to the plant rhizospheric soil bacterial community (Bao et al., 2022). Hence, replacing the inorganic with organic fertilizers has become a primary prerequisite for sustainable agriculture. Compared to the non-fertilizer treatment, the bioorganic fertilizer application improved ginseng plants' fresh and dry weight and shoot and root length (Shi et al., 2022). The ginseng-associated bacterial community composition shifted, leading to the enrichment of a specific subset of functional microbiota in the rhizosphere (Shi et al., 2022). Moreover, the addition of the bioorganic fertilizer altered the metabolomic profile of rhizospheric soils, which resulted in the decrease of phenolic acids, flavonoids, lipids, and alkaloid contents, thus stimulating specific plant-beneficial bacterial consortia involved in nutrient cycling and pathogen suppression (Shi et al., 2022).

As reported, the effects of cultivation are particularly pronounced on the bacterial communities, which in turn influence plant growth and health. Given the significant role of MP-associated microbiota, it is worthwhile studying these associations more comprehensively. Since wild plant microbiota may provide more beneficial functions than the one modified after cultivation, it is worth pursuing the study of microbiota from wild plants to promote modern crop yield (Martínez-Romero et al., 2020).

### 3.2 Endophytic bacteria improve MP productivity

Among the multiple types of symbiotic interactions, microbes and plants have mastered the ability to communicate. The microorganism recognizes and responds to the chemical signaling molecules produced by the plants, and once a plant–microbe relationship begins, both characters can sense their partner's physiology and coordinate their activities accordingly (Tripathi et al., 2022). Plant responses to the environment, including abiotic and biotic stress, are mediated to some extent by microbes. Hence, research on plant microbiomes may support targeted management approaches that are suited to the specific conditions of the field (Berg et al., 2020).

The increasing availability of microbiome data driven by advances in omics technologies has widened our understanding of the potential for microbiomes to enhance the productivity and sustainability of MPs (Berg et al., 2020). The development of the holobiont theory has unveiled a new source of genetic variation (i.e., the plant microbiome) (Gopal and Gupta, 2016). While strong candidate genes have not been identified, it is evident that specific plant traits act to recruit plant microbiomes (Gutierrez and Grillo, 2022). Therefore, the most strategic approach for crop improvement might be represented by breeding strategies aimed at selecting the specific traits that regulate microbiome assembly (Gutierrez and Grillo, 2022). In addition, as an integral part of the plant hologenome, the plant microbiome can be selected together with the plant genome, avoiding plant genetic manipulation (Gopal and Gupta, 2016). In this way, it would be possible to co-propagate the microbiome with the plant offspring in the new cultivation, together with a starter microbiome culture of keystone plant-beneficial microbiota from the initial soil, providing an opportunity for the plants to preferentially recruit the microbiota with which it had co-evolved (Gopal and Gupta, 2016).

Currently, another eco-friendly solution effective in alleviating issues due to cultivation issues is represented by the application of bioorganic fertilizer and complex microbial inoculants (Table 1). In general, microbiomes can be managed either directly by applying microbiome transplants, single microbes with beneficial properties, multifunctional microbial consortia, or microbiota-active metabolites, or indirectly by changing environmental conditions to produce a shift in their structure and function (Arif et al., 2020; Berg et al., 2021; Tabacchioni et al., 2021; Jansson et al., 2023; Shayanthan et al., 2022). The application of microbial fertilizer involves the utilization of organic matter for fermentation, together with the microbial inoculum made from beneficial strains (Liao and Xia, 2024). The microbial inoculum can be applied to the plants through drip irrigation, seed mixing, furrow, basal, hole, and spray application, and root irrigation (Chakraborty and Akhtar, 2021). The integration of plant-beneficial microorganisms such as nitrogen-fixing bacteria, phosphate-solubilizing microbes, PGPB, and arbuscular mycorrhizae became an environmentally benign alternative to the inorganic inputs (Gopal and Gupta, 2016). PGPB employ a wide range of mechanisms to directly promote plant growth and development (Narayanan and Glick, 2022). These mechanisms include (i) inorganic phosphate and potassium solubilization (Chen J. et al., 2021); (ii) the synthesis of siderophores that provide iron to plants (Ma et al., 2016); (iii) the production of phytohormones, like auxins, which promote plant cell elongation and proliferation; (iv) the release of 1-aminocyclopropane-1-caroxylate (ACC) deaminase, which lowers plant ethylene levels, thereby decreasing the inhibitory effects of various abiotic stresses (Orozco-Mosqueda et al., 2020). On the other hand, the indirect promotion of plant growth occurs when bacteria prevent or lessen the negative effects of pathogens or environmental stresses (Narayanan and Glick, 2022). Changes in climatic conditions such as rainfall, ambient CO_2_, and varying temperatures affect agriculture through countless constraints resulting in either low yields or sometimes death of the plants (Tripathi et al., 2022). Plant microbiome is known to respond ahead of its host plant to any environmental perturbation, regulating the gene expression of the host and modulating physiological responses and plant defense-related pathways (Gopal and Gupta, 2016; Tripathi et al., 2022). Microbiomes are effective in helping plants overcome salt and drought stress and in bioremediation, decreasing heavy metal stress, and eliminating harmful greenhouse gases (Tripathi et al., 2022; Ma et al., 2016); bacteria are also able to suppress phytopathogen infections through the production of antibiotics, fungal cell wall hydrolyzing enzymes, inhibiting volatile organic compounds (VOCs), and by inducing systemic resistance within plants (Pandey et al., 2019).

**Table 1:** Effects of plant growth-promoting rhizobacteria (PGPR) and bacterial endophytes on the secondary metabolism and yield of MPs.

**Medicinal plant species**	**Medicinal usage of the plant**	**Bacterial species**	**Application method**	**Effect of bacteria on plant secondary metabolism**	**References**
*Dracocephalum moldavica*	Traditionally used for its antioxidant and antibacterial EO	Arbuscular mycorrhizal fungi, nitrogen-fixing bacteria	Biofertilizer application with intercropping strategy	Increased EO content by 9.3%	Amiriyan Chelan et al., 2023
*Mentha arvensis* L.	Source of menthol used in medicine, flavoring	*Brevibacterium halotolerans* Sd-6, *Trichoderma harzianum*	Plantlets root were dipped in microbial suspensions	Enhanced oil content by 42.11% compared to control	Singh et al., 2019
*Mentha piperita*	Used for its aromatic, antibacterial and antioxidant properties	Beneficial rhizobacteria *Pseudomonas fluorescens* WCS417r, *P. putida* SJ04, *Bacillus subtilis* GB03	Individual and consortium inoculation in sterile vermiculite	Induced VOC emission and accumulation of phenolic compounds	Cappellari et al., 2017
*Thymus vulgaris*	Used for the treatment of fever, cough, cold, diabetes, and chest infections	Endophytic isolates *Bacillus licheniformis* T11r, *Bacillus velezensis* T13r	Soil inoculation with or without chemical fertilizers	Enhanced EO production, highest thymol concentration observed with endophytic bacteria and mineral fertilizer combination	Abdel-Hamid et al., 2021
*Ocimum basilicum*	Culinary and medicinal herbs possessing antiseptic, antioxidant, anti-stressor, antipyretic, and antimicrobial activities	PGP rhizobacteria *Pseudomonas putida* strain 41, *Azotobacter chroococcum* strain 5, *Azospirillum lipoferum* strain OF	Bacterial suspensions were mixed with sterile perlite	Increased shoot and root biomass, N, P, K content, and EO yield	Ordookhani and Zare, 2011
*Ocimum basilicum*	Culinary and medicinal herbs possessing antiseptic, antioxidant, anti-stressor, antipyretic, and antimicrobial activities	Rhizospheric *Bacillus* spp.	Seeds were mixed with bacterial suspensions	Increased fresh biomass yield, reduced root-knot disease severity, increased linalool, methyl chavicol, phenolic, and flavonoid contents	Gupta and Pandey, 2015
*Astragalus mongholicus*	Used in traditional medicine to treat tumors, hypertension, and diabetes	*A. mongholicus*-associated endophytic bacteria	Not applicable	Strong correlation with the accumulation of secondary metabolites	Li Y. et al., 2023
*Gentiana officinalis, G. siphonantha*	Used for its anti-inflammatory, antifungal, antihistamine, and antihepatotoxic activities	*Gentiana* spp.-associated endophytic bacteria	Not applicable	Metabolite content correlated with distinct bacterial endophytes	Hou et al., 2022
*Panax quinquefolius*	Used in traditional medicine to improve inflammatory processes, immune function, and response to exhaustion and stress	*Enterobacter* species	Not applicable	Positively correlated with saponin contents, enhanced conversion of plant metabolites into rare ones	Li R. et al., 2023
*Withania somnifera*	Used in Ayurveda for its anti-arthritic, anti-aging, anticancer, anti-inflammatory, immunoregulatory, chemoprotective, and cardioprotective activities	Endophytic isolates *Bacillus amyloliquefaciens* MPE20, *Pseudomonas fluorescens* MPE115	Seeds coating	Increased expression of MVA and MEP pathway genes; substantial increase in withaferin A, withanolide A and B; suppressed phytopathogen progression.	Mishra et al., 2018
*Atractylodes lancea*	Used in traditional medicine for its antibacterial, antiemetic, appetizer, digestive, diuretic, sedative, stomachic, and tonic properties	Endophytic bacterium *Acinetobacter* sp. ALEB16	Inoculation of meristem plant cultures	Increased EO content, stimulated MVA pathway gene expression due to bacterial emission of ABA and SA	Wang et al., 2015
*Bacopa monnieri*	Used for memory enhancement and anxiety reduction	Endophytic isolate *Bacillus subtilis*	Cuttings of *B. monnieri* were dipped in bacterial suspensions	Enhanced fresh and dry weight, increased Bacoside A content, upregulated phenylpropanoid and MEV/MVA pathway gene expression	Shukla et al., 2022
*Pelargonium graveolens*	Aromatic plants used in perfumery and aromatherapy	Endophytic isolates *Pseudomonas oryzihabitans* CB24, *Enterobacter hormaechei* CB7	Cuttings of *P. graveolens* were dipped in bacterial suspensions	Increased EO yield and fresh shoot biomass, increased concentration of geraniol, citronellol, and linalool	Deepa et al., 2024
*Papaver somniferum*	Source of opiates used in medicine	Endophytic isolates *Acinetobacter* sp. SM1B, *Marmoricola* sp. SM3B	Seeds soaking	Increased morphine, papaverine, and noscapine content, upregulated benzylisoquinoline alkaloid biosynthesis genes	Ray et al., 2019
*Lycoris radiata*	Contains alkaloids with anti-cholinesterase, antineoplastic, antimalarial, antiviral, antimicrobial, and anti-inflammatory properties	Bacterial isolates associated with *L. radiata*	Roots and bulbs of the plants were soaked in bacterial suspensions for 30 min	Increased alkaloid yield, attributed to indole-3-acetic acid (IAA) synthesis by bacteria	Liu et al., 2020
*Origanum syriacum* subsp. *sinaicum*	Culinary herb with antifungal, antibacterial, antioxidant, analgesic, anti-inflammatory, antispasmodic, anticancer, antiparasitic, insecticidal, nematicidal, and antimicrobial activities	Endophytic isolates *Bacillus* sp. SK1, *Bacillus* sp. SK2, *Serratia* sp. SK3, and *Serratia* sp. SK1	Seeds soaking	Increased EO yield, changes in EO components due to stimulation of nitrogen and phosphate uptake	Alraey et al., 2019
*Andrographis paniculata*	Traditional Asian MP with anti-inflammatory, anti-cholesterolemic, anti-allergenic, and antimicrobial properties	Bacterial isolate *Micrococcus luteus* (ASd6)	Plantlets were immersed in 30 ml of endophytic cultures for 30 min.	Significant increase in andrographolide content, especially with endophytes isolated from aerial parts	Kumari et al., 2023
*Artemisia annua*	Source of artemisinin used in malaria treatment	Seed-associated endophytes	Plantlets were dipped in microbial cultures for 30 min before planting in pots	Enhanced plant growth and artemisinin content	Tripathi et al., 2020

Individual inoculations or mixed inoculations yielded desirable results in some crops grown under certain soil and environmental conditions, demonstrating their efficacy in regulating both the structure and quantity of microbial communities in cultivated soils. The application of a biofertilizer composed of arbuscular mycorrhizal fungi and nitrogen-fixing bacteria, together with an intercropping strategy of Moldavian balm with fenugreek, represented an eco-friendly alternative to sole cropping and chemical fertilizer application, which was able to increase the EO content of Moldavian balm by 9.3% (Amiriyan Chelan et al., 2023). The EO yield was also enhanced in *Mentha arvensis* L. after the application of formulations of biofertilizers, including PGP strains and the fungus *Trichoderma harzianum*. Maximum EO content was observed in plants co-inoculated with *Brevibacterium halotolerans* Sd-6 and *T. harzianum* (42.11% more than control plants) in both pot experiments and field condition (Singh et al., 2019). Accordingly, the inoculation with beneficial rhizobacteria strains *Pseudomonas fluorescens* WCS417r, *P. putida* SJ04, and *Bacillus subtilis* GB03, individually or in consortia, caused a systemic induction of VOC emission and the accumulation of phenolic compounds in *M. piperita* plants (Cappellari et al., 2017). Moreover, the co-inoculation of PGP strains *Bacillus licheniformis* T11r and *B. velezensis* T13r, isolated from the roots of *Thymus vulgaris*, promoted the growth and enhanced the production of thyme EO, as compared to individually inoculated plants in the presence and absence of chemical fertilizer (Abdel-Hamid et al., 2021). Thymol concentration increased when the plants were treated with both a mineral fertilizer and the consortium of endophytic bacteria, suggesting that this inoculum could be used as a biofertilizer to enhance the growth of *T. vulgaris* as well as the content and quality of its EO (Abdel-Hamid et al., 2021). PGP rhizobacteria strains *P. putida* strain 41, *Azotobacter chroococcum* strain 5, and *Azospirillum lipoferum* strain of enhanced *Ocimum basilicum* growth, mostly increasing shoot and root fresh and dry weight, N, P, and K content, and EO yield (Ordookhani and Zare, 2011). Similarly, the fresh biomass yield of *Ocimum basilicum* was significantly higher when inoculated with PGP *Bacillus* spp., which also reduced the severity of the root-knot disease and induced the increase of linalool, methyl chavicol, and the phenolic and flavonoid contents with respect to untreated control (Gupta and Pandey, 2015). PGP strains can significantly increase productivity and reduce the amount of fertilizer required for the cultivation of economically important MPs, representing an efficient biotechnological tool for stimulating secondary metabolism in plants. However, the application of microbial inoculants/biofertilizers with PGP properties in agriculture has been proved to be often inconsistent (Qiu et al., 2019). The main reasons for failures are related to the plant-associated microbes' ability to exert their beneficial effects, due to incompatibilities with the host plant genotype and the growth environment, and the competition with other soil microorganisms (Nerva et al., 2022). Indeed, not all plant hosts and their associated microbiomes will respond in the same way to a particular introduced microbe, microbiome transplant, or metabolite (Berg et al., 2020).

Bacterial endophytes have closer relationships with their host plants; hence, their beneficial influences would likely last longer and be more efficient when inoculated in the same species (Ray et al., 2019). Endophytes–plant interactions can thus be exploited to optimize the production of bioactive compounds in MPs (Narayanan and Glick, 2022). In the association between endophytic bacteria and grapevine, a strong metabolic cross-talk induced the modulation of plant metabolism in response to bacterial inoculation (Lòpez-Fernàndez et al., 2016). It was observed that endophytic isolates could recolonize grapevine plantlets as endophytes, while leaving a metabolic signature; particularly, *Enterobacter ludwigii* EnVs6 induced the accumulation of hydroxycinnamic acids and flavonoids and a decrease in phytoalexins, suggesting the existence of a possible biological marker associated with endophytism (Lòpez-Fernàndez et al., 2016). The relationship between endophytic microbial communities' richness and secondary metabolite content of MPs was thoroughly investigated (Semenzato et al., 2023). In *Astragalus mongholicus*, the resident endophytic bacteria showed a strong correlation with the accumulation of secondary metabolites. Interestingly, the keystone species were significantly related to the presence of specific secondary metabolites, highlighting the fact that *A. mongholicus*-associated endophytes could be involved in the production of such compounds (Li Y. et al., 2023). The metabolite content of two *Gentiana* species (*G. officinalis* and *G. siphonantha*) grown in the same field was analyzed through high-performance liquid chromatography (HPLC), highlighting consistent differences between the two plants (Hou et al., 2022). The bacterial and fungal communities' composition was also determined, resulting in two distinct microbiomes. Correlation analysis between metabolites and endophytes showed that the content of loganic acid was significantly and positively correlated with the presence of specific endophytic fungi, while the content of gentiopicroside, swertiamarine, and sweroside was intertwined with the presence of distinct endophytic bacteria in the two species (Hou et al., 2022). Accordingly, the variation of the distribution of metabolites in four different tissues of *Panax quinquefolius*, MPs rich in saponins with pharmacological effects, was positively correlated with the presence of endophytic bacterial communities (Li R. et al., 2023). In particular, the presence of endophytes belonging to the genus *Enterobacter* was positively correlated with saponin content. Inoculation experiments confirmed their role in converting plant metabolites into rare ones, enhancing plants' pharmacological value (Wei et al., 2021).

**Figure 1:**
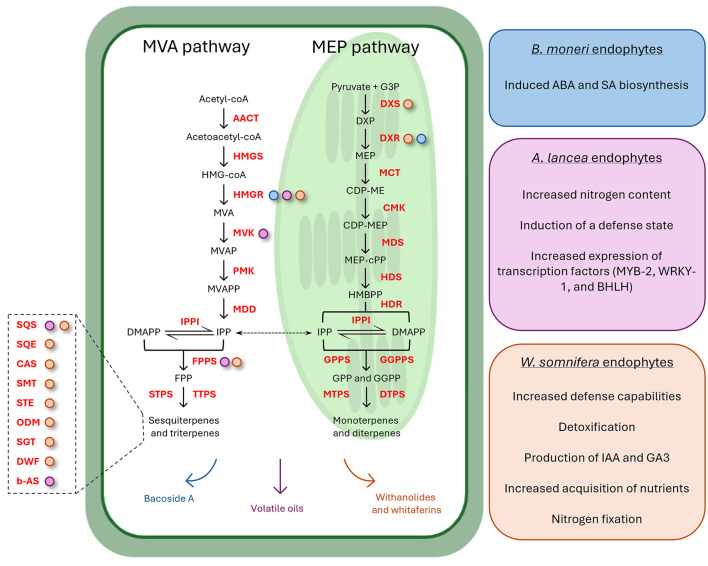
Schematic representation of endophytes' involvement in isoprenoid biosynthesis modulation. The central plant cell illustrates the MVA and MEP pathways from which monoterpenes, diterpenes, triterpenes, and sesquiterpenes are derived (compounds are reported in black, while enzymes catalyzing the reactions are in red). On the right, three colored boxes elucidate the underlying mechanisms contributing to the enhanced expression of isoprenoids biosynthetic genes in three medicinal plants: *Bacopa monnieri* (blue), *Atractylodes lancea* (pink), and *Withania somnifera* (orange). Arrows on the bottom of the plant cell are colored accordingly and indicate the specific metabolites whose concentration is increased after the endophytes' inoculation (Bacoside A, withanolides and withaferins, and volatile oils). Colored dots associated with enzymes denote their upregulated gene expression correlated with various endophyte-mediated mechanisms. The left magnification highlights additional enzymes in the triterpene biosynthetic pathway whose expression was upregulated after the endophytes' inoculation.

Endophytic bacteria are indeed able to influence the production of secondary metabolites and EOs in MPs, holding potential for many biotechnological applications (Chamkhi et al., 2021). Compared to the large body of literature showing how plant secondary metabolites can shape the plant–microbiome structure, our understanding of the effects of the microbiome on plant secondary metabolites, including their mechanism of action, remains quite limited (Pang et al., 2021). The plant–endophyte interactions might induce significant changes in the metabolome of the holobiont due to the induction of host metabolism by the endophytes. Additionally, some metabolic pathways may be shared between the two partners; endophytic bacteria could produce bioactive metabolites using plant-derived precursors, and/or vice versa. For some MPs, investigations have shown that the plant microbiomes could influence host plants' productivity of important medicinal components such as alkaloids, steroids, terpenoids, flavonoids, glycosides, and antioxidant molecules (Ferreira et al., 2023; Ogbe et al., 2020). The inoculation of axenic *Echinacea purpurea* plantlets grown *in vitro* using a bacterial consortium composed of *E. purpurea*-associated endophytes resulted in the enhancement of VOC emission and accumulation of chicoric acid (Maggini et al., 2019a,b). Various studies have reported the ability of microbes to enhance the isoprenoid biosynthesis, as depicted in Figure 1.

Isoprenoids, also known as terpenoids, are essential for various biological functions, including the synthesis of hormones, sterols, and quinones, as well as for membrane stability and electron transport (Holstein and Hohl, 2004). The mevalonate (MVA) and methylerythritol phosphate (MEP) pathways are two distinct biosynthetic routes for the production of isoprenoids. These pathways are present in both prokaryotes and eukaryotes, but their distribution and utilization differ significantly between bacteria and plants. The MVA pathway is primarily found in eukaryotes, while the MEP pathway is the predominant route in bacteria (Marshall et al., 2023). In plants, the MVA pathway is localized in the cytoplasm and is responsible for the synthesis of sterols, sesquiterpenes, and certain other isoprenoids, while the MEP pathway is localized in plastids, where it contributes to the synthesis of essential isoprenoids like chlorophylls, carotenoids, and plastoquinones (Zeng and Dehesh, 2021). This pathway is distinct from the MVA pathway in both its intermediates and enzymes, making it a potential target for the development of novel antibiotics and biotechnological engineering (Pérez-Gil and Rodríguez-Concepción, 2013). *Withania somnifera* (L.) Dunal is an important MP producing diverse therapeutically important compounds. The application of two bacterial endophytic isolates (*Bacillus amyloliquefaciens* MPE20 and *P. fluorescens* MPE115) suppressed the progression of the phytopathogen *Alternaria alternata* growth, significantly reducing disease severity (Mishra et al., 2018). In addition, MVA and MEP biosynthesis gene expression was upregulated in endophytes-treated plants under pathogenic stress, with a consequent substantial increase of therapeutically important secondary metabolite content, such as withaferin A and withanolide A and B (Mishra et al., 2018). Similar results were obtained by Pandey et al. (2018). Endophytes-inoculated *Withania* plants had 20%−232% higher withanolide content in leaves compared to non-inoculated endophyte-free control plants (Pandey et al., 2018). Surprisingly, some endophytes induced the synthesis of withaferin A in roots, which is abundantly synthesized in leaves and absent or present in traces in roots of some varieties of *W. somnifera* (Pandey et al., 2018). Endophyte inoculation modulated the expression of genes of withanolide biosynthesis (which involves both MVA and MEP pathways) both in leaves and roots, suggesting a strong effect on plant gene expression despite the plant compartment (Pandey et al., 2018). HMGR-dependent MVA pathway was also involved in the accumulation of the EO produced by *Atractylodes lancea* plants when inoculated with the endophyte *Acinetobacter* sp. ALEB16. The endophytic bacterium did not affect plantlet growth, suggesting its involvement in stimulating the secondary metabolite biosynthesis of the plantlets and not the primary one (Wang et al., 2015). In particular, the transcriptional and enzymatic activities of biosynthetic genes of the MVA pathway were due to the bacterial emission of abscisic acid and salicylic acid (secondary metabolism elicitors) (Wang et al., 2015). Similarly, a promising effect of bacterial endophytes was also observed on the Bacoside A content of *Bacopa monnieri* (L.) Pennell, an economically important MP used for improving memory, attenuating mental deficits, and reducing anxiety (Shukla et al., 2022). The application of single isolates or endophytic consortia significantly enhanced the fresh weight, dry weight, and Bacoside A content of the plant (Shukla et al., 2022). The gene expression analysis revealed the significant upregulation of phenylpropanoid biosynthetic genes in the case of plants inoculated with *B. subtilis*, together with an overall increase in MEV and MVA pathway gene expression, which provided a pool of substrates for the synthesis of higher amounts of bacosides (Shukla et al., 2022). In a recent study, endophytic isolates with PGP activity enhanced plant growth and development of *Pelargonium graveolens*, while also increasing the production of volatile secondary metabolites of industrial importance (Deepa et al., 2024). The isolates *Pseudomonas oryzihabitans* CB24 and *Enterobacter hormaechei* CB7 increased EO yield and enhanced the fresh shoot biomass with respect to control plants (Deepa et al., 2024). The concentration of EO compounds, such as geraniol, citronellol, and linalool content, increased after the inoculation, due to endophytes' ability to alter or modulate plant secondary metabolic pathways (Deepa et al., 2024). Endophytes affecting the host metabolism at the gene expression level, but having different modes/targets of action, could be combined to complement the inability of one endophyte to upregulate certain genes, thus improving the biosynthesis of secondary metabolites (Ray et al., 2019). This was observed for endophytic bacteria *Acinetobacter* sp. SM1B and *Marmoricola* sp. SM3B, which could upregulate most of the genes of benzylisoquinoline alkaloids biosynthesis, but not the entire pathway, in alkaloidless *Papaver somniferum*. The content of morphine, papaverine, and noscapine in the capsule of plants inoculated with both endophytes significantly increased to a greater extent than that of single inoculations and control plants (Ray et al., 2019).

In some cases, the accumulation of secondary metabolites in plants could reflect the PGP activity of the inoculants. Plants of the Amaryllidaceae family are unique producers of alkaloids, known to have various medicinal activities, such as anti-cholinesterase, antineoplastic, antimalarial, antiviral, antimicrobial, and anti-inflammatory ones (Liu et al., 2020). Bacterial endophytes could significantly increase the concentration of the alkaloids of interest in *Lycoris radiata* (Liu et al., 2020). The mechanisms involved in the enhancement of the total yield of alkaloids were attributed to the bacterial synthesis and secretion of indole-3-acetic acid. This hormone can enhance root development and nutrient uptake of the host plants, benefitting the plant's primary metabolism and stimulating plant defense responses; as a consequence, the enhancement of secondary metabolism should also be expected, thereby leading to the increment of the production of the valuable alkaloids (Liu et al., 2020). The PGP bacterial endophytic isolates *Bacillus* sp. SK1, *Bacillus* sp. SK2, *Serratia* sp. SK3, and *Serratia* sp. SK1, associated with *Origanum* plants obtained from a wild habitat, increased the amounts of EOs yield in pot-grown *Origanum* plants when compared with untreated control plants (Alraey et al., 2019). Carvacrol, γ-terpinene, and p-cymene were the major components, whose concentrations (%) varied among the different treatments (Alraey et al., 2019). The changes in the EOs amount and constituents were attributed to the PGP activity of those strains; indeed, the stimulation of nitrogen and phosphate uptake induced by the endophytes is reported to play a key role in EOs biosynthesis as these elements are involved in terpenoid biosynthesis (Alraey et al., 2019).

All anatomical parts of the plant are virtually colonized by bacterial endophytes, which seem to be well adapted to their niche of isolation (Compant et al., 2011). Such niche specificity might be linked to the functional behavior of the endophytes (Castronovo et al., 2020; Chiellini et al., 2014). Endophytes associated with plant parts where the production of secondary metabolites is higher could possess a greater capacity to promote secondary metabolite synthesis when used in inoculation experiments. This suggests that selecting endophytes from these specific niches may enhance the overall production of pharmacologically relevant compounds in the host plant. The annual herbaceous plant *Andrographis paniculata*, used in the traditional medical systems of many Asian nations, is a valuable source of distinctive bioactive secondary metabolites with anti-inflammatory, anti-cholesterolemic, anti-allergenic, and antimicrobial properties (Kumari et al., 2023). Isolation of bacterial endophytes was carried out from various plant tissues, namely root, stem, leaves, and seeds of *A. paniculata*, and bacteria were then used for inoculation experiments (Kumari et al., 2023). According to the HPLC analysis, there was a significant increase in andrographolide content in inoculated plants in comparison to the control plants (Kumari et al., 2023). This effect was more evident for endophytes isolated from the aerial part, clearly indicating the niche specificity of such strains (Kumari et al., 2023).

Seed-associated endophytes are known to stimulate seed germination, promote seedling growth and resilience, and are vertically transmitted, providing subsequent plant generations with beneficial symbionts (Nelson, 2018; Chen et al., 2018). *Artemisia annua* seed-associated endophytes were used to inoculate pot-grown *A. annua* plants. Bacterial isolates enhanced plant growth and artemisinin content over control plants, highlighting the potential of seed-endophytic strains in enhancing plant growth as well as the secondary metabolite content of MPs (Tripathi et al., 2020).

**Figure 2:**
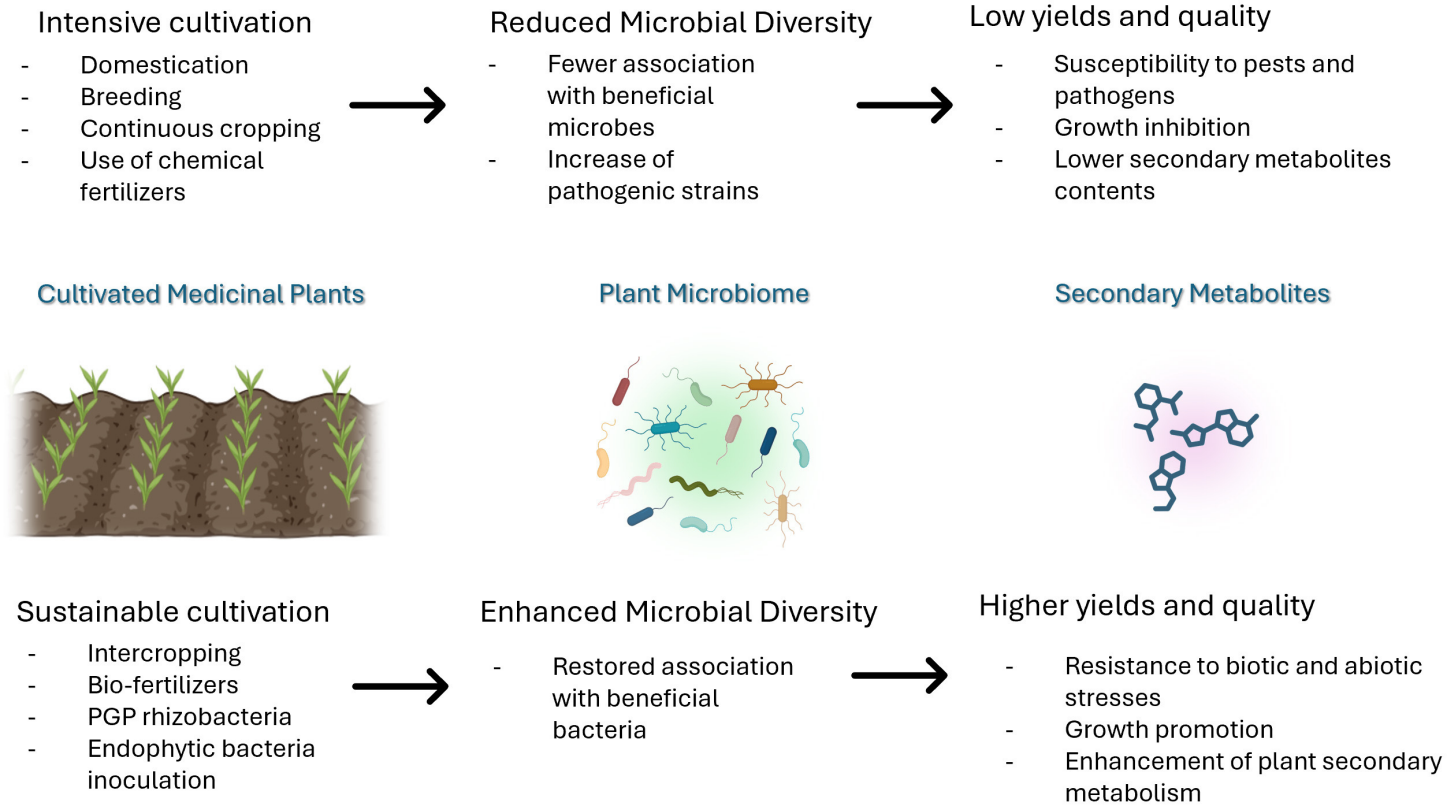
The effect of cultivation practices on MP-associated microbiomes and plant secondary metabolism.

The effect of bacterial endophytes on EO content and composition was observed in many MPs, in standard conditions or when subjected to abiotic or biotic stresses (Dehghani Bidgoli et al., 2019). However, the still limited knowledge of the mechanisms underlying plant ability to control its associated microbial communities and how members of microbial consortia interact with one another strongly limit their exploitation in agriculture (Nerva et al., 2022). Endophytic microorganisms exhibit tissue specificity, and their establishment and functionality within the host are influenced by several factors such as tissue type, host genotype, and surrounding environmental conditions. The development of successful endophyte application technologies relies heavily on enhancing our understanding of their mechanisms of entry and colonization within the plant (Liao and Xia, 2024). Leveraging the relationship between plants and endophytes is crucial for advancing sustainable development, and extensive research is necessary to either confirm or refute this hypothesis. Therefore, in-depth future studies are essential to gain a better understanding of endophytes within their hosts, thereby advancing the viability of endophyte-assisted biological applications, particularly in field settings (Figure 2) (Tripathi et al., 2022).

### 3.3 Obtaining bioactive molecules directly from endophytes

In addition to their ability to modulate the plant's secondary metabolism, endophytic bacteria encompass a high potential for the synthesis of an ample range of novel secondary metabolites within or without host plants (Table 2). These compounds belong to various chemical groups like xanthones, terpenoids, phenols, steroids, benzopyranones, isocoumarins, chinones, cytochalasins, tetralones, and enniatines (Alvin et al., 2014). Endophytic bacteria represent an almost untapped source of molecules with biological activities, including antioxidant, anticancer, immunosuppressive, and antidiabetic properties, as well as antifungal, antibacterial, antiviral, and/or nematicidal ones (Tripathi et al., 2022). Given their immense therapeutic potential, these secondary metabolites offer significant benefits to humanity, such as reduced costs, a lower carbon footprint, and the conservation of endangered plant species (Narayanan and Glick, 2022; Semenzato et al., 2024). If in the past, the research on traditional MPs mainly focused on their bioactive compounds, now this trend has shifted toward the exploitation of MP-associated endophytes (Tanvir et al., 2017). MPs represent the primary choice for isolating bioactive molecules-producing endophytes; indeed, a plant species having a unique ethnobotanical history and so a unique environmental habitat for its microbiota might ensure greater possibility to isolate microbes with potential pharmacological and biotechnological applications (Strobel and Daisy, 2003). It is believed that the phytochemical biosynthesis functions of endophytes were obtained by the horizontal gene transfer through their long-term evolution within their hosts (Chen Y. et al., 2021). In contrast, some other studies showed that the secondary metabolite biosynthetic genes in some endophytes were not homologous with their hosts, and some genes were even absent in the host genomes, indicating that the phytochemical biosynthetic pathways of endophytes might have evolved independently (Chen Y. et al., 2021).

**Table 2:** Metabolites produced by bacterial endophytes and their biological activities.

**Medicinal plant species**	**Endophytic isolates**	**Metabolites produced**	**Biological activity of endophyte extracts**	**References**
*Pinellia ternata, Lycium chinense, Digitalis purpurae, Leonurus heterophyllus, Bletilla striata, Belamcanda chinensis, Pinellia pedatisecta, Taxus yunnanensis*	*Moraxella, Pseudomonas, Klebsiella, Enterobacter, Citrobacter, Erwinia, Achromobacter, Methylobacterium, Paracoccus, Sphingomonas, Microbacterium, Paenibacillus, Bacillus, Staphylococcus* spp.	Cytotoxic, antibacterial, antifungal polyketides and non-ribosomal peptides (gene-inferred)	Cytotoxic, antibacterial, antifungal activity against multiple myeloma RPMI-8226 cells, *S. aureus, E. coli, C. albidus*, and *C. albicans*	Miller et al., 2012
*Millettia pachycarpa*	*Paenibacillus peoriae* IBSD35	Peoriaerin II (gene-inferred)	Broad-spectrum antimicrobial activity against *S. aureus, E. coli*, and *C. albicans*	Ngashangva et al., 2021
*Solanum nigrum*	*Bacillus* sp.	Bacillibactin, Fengycin, diterpenoids, terpene glycosides, alpha-amino acids, oligopeptides (gene-inferred)	Anticancer activity through stabilization of FGF protein-ligand complexes	Maumela and Serepa-Dlamini, 2024
*Mimosa pudica*	*Staphylococcus caprae* LL2, *Priestia megaterium* LL1	Phenols, flavonoids, terpene, siderophore, polyketide, non-ribosomal peptide, lanthipeptide, betalactone (gene-inferred)	Antioxidant	Akter et al., 2022
*Calotropis procera*	*Bacillus siamensis, B. amyloliquefaciens*	Phenolic and flavonoid compounds	Antibacterial activity against *E. coli, K. pneumoniae, S. agalactiae, S. typhi, S. marcescens*, and *S. aureus*	Hagaggi and Mohamed, 2020
*Urtica dioica*	*Bacillus* spp.	Polyphenols including caffeic acid and chlorogenic acid	Antioxidant and antimicrobial activity	Marchut-Mikołajczyk et al., 2023
*Viola odorata*	*Enterobacter, Microbacterium, Pseudomonas, Rhizobium, Streptomyces* spp.	Phenolic compounds	Antioxidant activity with free radical scavenging abilities	Salwan et al., 2023
*Emilia sonchifolia*	*Bacillus subtilis* ES1	Hexadecanoic acid, heptatriacotanol, surfactin	Anti-inflammatory activity	Urumbil and Anilkumar, 2021
*Artemisia nilagirica*	*Chromobacterium violaceum* WVAT6, *Burkholderia* sp. WYAT7	Not available	Antibacterial activity against human pathogens	Ashitha et al., 2019
*Crinum macowanii*	Raoultella ornithinolytica	Not available	Antibacterial activity against *K. pneumoniae, E. coli*, and *P. aeruginosa*	Sebola et al., 2019
*Dicoma anomala*	*Stenotrophomonas* sp. strain MHSD20, *Enterobacter* sp. strain MHSD22, *Staphylococcus* sp. strain MHSD26, *Bacillus* sp. strain MHSD28, *Stenotrophomonas* sp. strain MHSD12, *Bacillus* sp. strain MHSD14, *Pantoea* sp. strain MHSD15	Benzyl benzoate; cyclohexasiloxane, dodecamethyl; 9-octadecenamide (Z); hexadecane; pyrrolo[1,2-a] pyrazine-1,4-dione, hexahydro-3-(2-methylpropyl); octasiloxane, 1, 1, 3, 3, 5, 5, 7, 7, 9, 9, 11, 11, 13, 13, 15, 15-hexadecamethyl-; heptasiloxane, hexadecamethyl; dibutyl phthalate; indole; heptacosane; 9-eicosene; benzoic acid, 4-ethoxy-, ethyl ester; triclosan	Antibacterial activity against *E. coli, B. cereus, S. aureus, P. aeruginosa, K. oxytoca*	Makuwa and Serepa-Dlamini, 2021
*Moringa oleifera, Artemisia afra, Aloe vera*	*Pseudomonas fulva* I3–I5, *Enterobacter cloacae* I4	Alkanes, esters, alcohols, ketones, L-proline	Antibacterial and antifungal activity, antioxidant properties	Nchabeleng et al., 2024
*Fagonia indica*	*Stenotrophomonas maltophilia*	Esters, fatty acids, alcohols, aldehydes, terpenes, amides	Broad-spectrum antibacterial activity, with strong affinity for receptor proteins involved in pathogenic bacterial outer membrane biosynthesis	Rahman et al., 2022
*Alectra sessiliflora*	*Lysinibacillus* sp. AS_1, *Bacillus* sp. AS_3	Octacosane, tetracosane, eicosane, phenol derivatives	Antibacterial and cytotoxic activity against cancer cell lines	Maela et al., 2022
*Artemisia absinthium*	*Pseudomonas aeruginosa* sp. SD01	2-aminoacetophenone, pyrrolo[1,2-a]pyrazine-1,4-dione, phenazine, 2-phenyl-4-cyanopyridine	Antibacterial activity against multidrug-resistant *S. aureus* and apoptosis induction in MCF7 tumor cells	Damavandi et al., 2023

One possible approach aimed at discovering endophytic secondary metabolites is represented by genome mining. Genome mining offers significant advantages in the identification and characterization of secondary metabolites from endophytes. This approach allows researchers to predict the presence of biosynthetic gene clusters (BGCs) responsible for metabolite production by analyzing genomic data (Vitali et al., 2023). One of the primary benefits is its predictive power, enabling the discovery of novel compounds that may not be identifiable through traditional methods. By identifying potential BGCs, researchers can focus their efforts on strains with predicted biosynthetic capabilities, reducing the need for extensive chemical screenings (Pitakbut et al., 2022). However, genome mining also has its limitations. One major drawback is the reliance on existing databases of known BGCs, which can lead to incomplete or inaccurate predictions if novel or highly divergent metabolites are produced that are not represented in these databases (Yamada et al., 2015). Data generated from genome mining always need to be experimentally validated, involving further isolation, characterization, and testing of the predicted metabolites (Sekurova et al., 2019). Indeed, most of the metabolites obtained from endophytic bacteria have been characterized after isolating bacteria and growing them *in vitro* (Brader et al., 2014). When combined with traditional methods of metabolite isolation and characterization, genome mining offers a valuable means to explore and exploit the biosynthetic potential of endophytes. Polyketide synthases (PKS) and non-ribosomal peptide synthetases (NRPS) are two classes of large, multifunctional enzyme complexes responsible for the biosynthesis of a vast array of structurally diverse and biologically active natural products (Donadio et al., 2007). These compounds include many clinically important antibiotics, immunosuppressants, anticancer agents, and other therapeutics (Zhang et al., 2014). The PKS and NRPS pathways are particularly notable for their ability to produce complex molecules with intricate structures that are often difficult to synthesize chemically. Their modular nature and ability to incorporate various building blocks make them valuable tools in biotechnology and drug development and for the discovery and synthesis of novel therapeutics (Beck et al., 2020). With only a few endophytic NRPS- and PKS-coding genes identified and functionally characterized, there is a significant opportunity to characterize novel secondary metabolite pathways and thus increase our understanding of bacterial biosynthetic routes (Miller et al., 2012). Bacterial endophytes belonging to 14 different genera and isolated from eight species of plants used in Traditional Chinese Medicine, were surveyed for the presence of PKS and NRPS genes (Miller et al., 2012). Many unique and previously uncharacterized biosynthetic pathways were detected, and their involvement in the production of novel bioactive metabolites was assessed (Miller et al., 2012). Bacterial ethyl acetate extracts showed cytotoxic, antibacterial, or antifungal activity against multiple myeloma RPMI-8226 cells, *Staphylococcus aureus, Escherichia coli, Candida albidus*, and *C. albicans*, supporting the gene-inferred biosynthetic potential of these isolates (Miller et al., 2012). In a more recent study, the combination of genomic data with LC-MS/MS analysis and molecular networking, led to the identification of a highly potent and broad-spectrum antimicrobial compound, from the Gram-positive endophyte *Paenibacillus peoriae* IBSD35, isolated from the stem of *Millettia pachycarpa* Benth (Ngashangva et al., 2021). The purified fermentation sample, obtained through preparative HPLC, exhibited broad-spectrum antimicrobial activity against *S. aureus, E. coli*, and *C. albicans* (Ngashangva et al., 2021). From genome sequence data, a NRPS biosynthetic gene cluster was predicted, which is believed to encode the compound Peoriaerin II, holding promise for further drug development against antimicrobial-resistant pathogens (Ngashangva et al., 2021). *Solanum nigrum* is used in traditional medicine to treat conditions such as tumors, asthma, inflammation, and bacterial and viral infections (Maumela and Serepa-Dlamini, 2024). Whole genome sequencing and assembly allowed the identification of a leaves-associated bacterial endophyte, identified as a novel *Bacillus* subspecies (Maumela and Serepa-Dlamini, 2024). Genome annotation using anti-SMASH revealed the presence of bacillibactin and Fengycin BGCs, which are commonly produced by *Bacillus* species (Maumela and Serepa-Dlamini, 2024). Further analysis using LC-MS confirmed the presence of a wide range of secondary metabolites, including diterpenoids, terpene glycosides, alpha-amino acids, and oligopeptides, having potential anticancer activity (Maumela and Serepa-Dlamini, 2024).

Bacterial endophytes associated with MPs are believed to produce the same bioactive metabolites or ones that replicate the beneficial effects of the host plants (Polito et al., 2022). Bacterial endophytes have demonstrated antioxidant activity through the production of phenols and flavonoids, which are commonly found in MP metabolomes (Goulart et al., 2019). The antioxidant activity of ethyl acetate extracts obtained from bacterial endophytes isolated from fresh and healthy MPs collected at the University of Chittagong was evaluated (Akter et al., 2022). The endophyte *Staphylococcus caprae* was characterized by the highest total phenolic content and total flavonoid content, suggesting its potential as a source of natural antioxidant compounds (Akter et al., 2022). To investigate the biosynthetic gene clusters and the associated bioactive secondary metabolites from the isolated bacterial strains, the webserver tool anti-SMASH was used (Blin et al., 2021). The analysis revealed 13 genomic regions coding for different secondary metabolites across five isolates, with terpene, siderophore, and type III polyketide synthase regions being the most common, followed by NRPS, lanthipeptide, and betalactone regions (Akter et al., 2022). Among the isolates analyzed, the *Priestia megaterium* strain showed the highest number of metabolite regions, positioning it as a potent source of bioactive compounds (Akter et al., 2022). The bacterial endophytes *Bacillus siamensis* and *B. amyloliquefaciens* associated with the MP *Calotropis procera* were chosen as models to compare their production of bioactive secondary metabolites with that of the host plant. Interestingly, in most cases, the extracts from these endophytic bacteria contained higher concentrations of secondary metabolites than those found in the host plant itself (Hagaggi and Mohamed, 2020). The ethyl acetate, methanol, and aqueous extracts of *C. procera* plants and of the bacterial endophytes were tested against *E. coli, Klebsiella pneumoniae, Streptococcus agalactiae, Salmonella typhi, Serratia marcescens*, and *S. aureus* (Hagaggi and Mohamed, 2020). Interestingly, the extracts of both *B. siamensis* and *C. procera* showed the broadest antibacterial spectrum (Hagaggi and Mohamed, 2020). The effectiveness of these extracts was attributed to the high phenolic and flavonoid content, as indicated by significant positive correlations between those metabolite contents and the antibacterial activity against all tested pathogenic bacteria (Hagaggi and Mohamed, 2020). *Urtica dioica* has long been recognized for its beneficial health effects, largely attributed to its high polyphenolic content. Three Gram-positive endophytic *Bacillus* spp. isolated from *U. dioica* could synthesize phenolic compounds *in vitro*. Gas chromatography–mass spectrometry (GC/MS) analysis further revealed that the total polyphenol content included caffeic acid and chlorogenic acid, alongside some lipid compounds (Marchut-Mikołajczyk et al., 2023). Accordingly, methanol extracts from endophytic isolates associated with *Viola odorata* roots showed free radical-scavenging abilities due to the high phenolic content of the extracts (Salwan et al., 2023). In particular, isolates with higher phenolic content also exhibited greater antioxidant activity, highlighting the correlation between antioxidants and phenolics (Salwan et al., 2023). Molecular identification of the promising isolates revealed various species from the genera *Enterobacter, Microbacterium, Pseudomonas, Rhizobium*, and *Streptomyces* (Salwan et al., 2023). To fully exploit the potential of endophytic bacteria for polyphenol production, further studies are needed to elucidate the mechanisms of their biosynthesis. Anyway, these compounds are known to inhibit microbial growth by damaging cell walls and altering metabolic pathways, posing a significant challenge to achieving high-efficiency microbial production of these chemicals (Marchut-Mikołajczyk et al., 2023).

The anti-inflammatory properties of *Emilia sonchifolia* (Linn.) DC, an erect herbaceous plant from the Asteraceae family, were effectively mimicked by the ethyl acetate extract of its bacterial endophytes (Urumbil and Anilkumar, 2021). *In vitro* studies, which measured egg albumin denaturation and heat-induced hemolysis of red blood cells, demonstrated that the extract from the endophyte *Bacillus subtilis* ES1 exhibited significantly high anti-inflammatory activity (Urumbil and Anilkumar, 2021). This was further supported by strong inhibition of cyclooxygenase, lipoxygenase, and myeloperoxidase enzymes. The *in vivo* analysis revealed that administering the bacterial extract significantly reduced inflammation-induced mice paw thickness, indicating notable anti-inflammatory effects (Urumbil and Anilkumar, 2021). The extract's composition included known anti-inflammatory compounds, such as hexadecanoic acid, heptatriacotanol, and surfactin (Urumbil and Anilkumar, 2021).

Concerning the antibacterial activity of endophytes-derived compounds, many promising results have been obtained. *Artemisia nilagirica* is extensively used in ayurvedic and homeopathic medicine for its antibacterial properties (Ashitha et al., 2019). The bacterial endophytes *Chromobacterium violaceum* WVAT6 and *Burkholderia* sp. WYAT7 showed maximum antagonistic activity against human pathogens (Ashitha et al., 2019). The ethyl acetate extract of sonicated bacterial culture exhibited the highest inhibitory activity against most of the pathogens tested, suggesting the presence of antimicrobial metabolites inside the bacterial cells (Ashitha et al., 2019). Accordingly, the endophytic crude extracts obtained by bacteria isolated from *Crinum macowanii* Baker showed antibacterial activity against human pathogenic strains, with *Raoultella ornithinolytica* extract exhibiting a significant growth inhibition of *K. pneumoniae, E. coli*, and *P. aeruginosa* strains (Sebola et al., 2019). Consistently, *Dicoma anomala*-associated bacterial endophytes crude extracts were able to inhibit the growth of the pathogenic strains *E. coli, B. cereus, S. aureus, P. aeruginosa*, and *Klebsiella oxytoca* (Makuwa and Serepa-Dlamini, 2021). Different classes of compounds known for their antimicrobial, antioxidant, and anti-inflammatory activities were identified from the ethyl acetate crude extracts of those bacterial endophytes (Makuwa and Serepa-Dlamini, 2021). *Moringa oleifera*-, *Artemisia afra*-, and *Aloe vera*-associated endophytes were tested for their inhibitory activity against *Mycobacterium bovis*. The pathogen was sensitive to the secondary metabolites produced by endophytes *Pseudomonas fulva* I3–I5 and *Enterobacter cloacae* I4 (Nchabeleng et al., 2024). The GC-MS analysis revealed the production of alkanes, esters, alcohols, ketones, and other organic molecules with known antifungal, antibacterial, and antioxidant properties, such as hexadecane and L-proline (Nchabeleng et al., 2024). Endophytic bacterial strains isolated from *Fagonia indica* exhibited a broad-spectrum antibacterial activity against human pathogenic bacteria, showing antagonistic potential against all tested Gram-positive and Gram-negative pathogenic strains (Rahman et al., 2022). Notably, the endophytic bacterium *Stenotrophomonas maltophilia* extract induced the highest inhibitory activity against *Staphylococcus caseolyticus* and *Acinetobacter baumannii* (Rahman et al., 2022). To identify the compounds responsible for these pharmacological activities, the extracts were further analyzed using GC-MS, revealing the presence of esters, fatty acids, alcohols, aldehydes, terpenes, and amides (Rahman et al., 2022). Notably, while the major constituents were identified, some compounds with high concentrations were not matched in the existing compound library, suggesting they may be novel (Rahman et al., 2022). Molecular docking analysis further revealed that these compounds show affinity to broad-spectrum receptor proteins involved in the biosynthesis of the outer membrane of pathogenic bacterial cells (Rahman et al., 2022).

In some instances, the antimicrobial activity of endophytes derived from MPs has also been linked to effective cytotoxic activity against tumor cell lines. *Alectra sessiliflora* is a MP that grows across Sub-Saharan Africa, China, India, and the Philippines (Maela et al., 2022). Plant extracts of *A. sessiliflora* exhibited antibacterial activity against pathogens such as *S. aureus, P. aeruginosa, E. coli, B. pumilus*, and *Shigella dysenteriae* at minimum inhibitory concentration values ranging from 3.13 to 25 mg/ml (Maela et al., 2022). The antimicrobial activity of bacterial endophytes associated with *A. sessiliflora* was also tested. *Lysinibacillus* sp. strain AS_1 exhibited broad antibacterial activity against the pathogenic strains with MIC values ranging from 4 to 8 mg/ml, while *Bacillus* sp. strain AS_3 displayed MIC of 0.25 mg/ml (Maela et al., 2022). The antitumor potential of the bacterial endophyte crude extracts was further evaluated against three human cancer cell lines, showing cytotoxic effects against all three (Maela et al., 2022). The chemical composition of the crude extracts was further analyzed using GC, identifying 80 compounds across various chemical groups. Notably, several alkane compounds, such as octacosane, tetracosane, and eicosane, secreted by *Bacillus* sp. AS_3, have been reported as potential inhibitors against various cancer cells, including cervical carcinoma, breast carcinoma, and human embryonic lung cells (Maela et al., 2022). Additionally, phenol and phenol derivatives, such as 4-mercaptophenol, phenol, 2,2-bis(1,1-dimethyl), and 2,4-di-tert-butylphenol, were identified in some bacterial extracts, which are known for their significant therapeutic properties, including anticancer, antibacterial, antiseptic, and anti-inflammatory effects (Maela et al., 2022). Ethyl acetate extracts of 10 bacterial endophytes isolated from *Artemisia absinthium* exhibited antibacterial activity against human pathogenic bacteria, each producing distinct inhibition zones (Damavandi et al., 2023). Among these, the isolate *P. aeruginosa* sp. SD01 demonstrated the most potent antibacterial activity, particularly against *S. aureus* multidrug-resistant strains, which showed signs of lysis, pore formation on the cell surface, and leakage of intracellular components (Damavandi et al., 2023). The bioactive secondary metabolites from SD01 also induced significant apoptosis in MCF7 tumor cells, leading to programmed cell death without any signs of necrosis (Damavandi et al., 2023). The observed activities were attributed to the presence of important bioactive secondary metabolites, such as 2-aminoacetophenone, pyrrolo[1,2-a]pyrazine-1,4-dione, phenazine, and 2-phenyl-4-cyanopyridine, with known antimicrobial and anticancer activities (Damavandi et al., 2023).

## 4 Discussion and conclusion

This review highlights the often-overlooked role of microbial endophytes, particularly bacteria, in enhancing the productivity of MPs and serving as “factories” for natural compounds. These endophytes hold significant potential for the development of pharmaceutical drugs, blending traditional medicinal knowledge with modern healthcare practices. Such integration is not only essential for cultural preservation but also for expanding therapeutic options, especially in resource-limited settings where access to conventional medicine may be limited. The study of MPs and their associated microbiomes fosters interdisciplinary research, bridging fields such as pharmacology, ethnobotany, and conservation biology together with microbiology. The integration of plant breeding, precision farming, agricultural management, and microbiome research represents a robust strategy for enhancing sustainable crop production in a rapidly changing world. Advances in microbiome management, including the engineering of environmental microbiomes, promise a future where toxic chemicals in agriculture are replaced by more sustainable alternatives. However, the application of microbial consortia in open agricultural systems remains a challenge due to the complex nature of microbe–plant interactions (Gopal and Gupta, 2016; Neuhoff et al., 2024). Continued research is essential to identify the most relevant bacterial species and develop effective consortia. By addressing these challenges, we can unlock the full potential of microbial endophytes, contributing to sustainable agriculture and improved public health on a global scale.

## Footnotes

^1^ https://www.ecfr.gov/current/title-21/chapter-I/subchapter-B/part-182
